# Virtual Sensor for On-Line Hardness Assessment in TIG Welding of Inconel 600 Alloy Thin Plates

**DOI:** 10.3390/s24113569

**Published:** 2024-06-01

**Authors:** Jacek Górka, Wojciech Jamrozik, Bernard Wyględacz, Marta Kiel-Jamrozik, Batalha Gilmar Ferreira

**Affiliations:** 1 Department of Welding Engineering, Silesian University of Technology, Konarskiego Str. 18a, 44-100 Gliwice, Poland; bernard.wygledacz@polsl.pl; 2 Department of Fundamentals of Machinery Design, Silesian University of Technology, Konarskiego Str. 18a, 44-100 Gliwice, Poland; 3 Department of Biomaterials and Medical Devices Engineering, Silesian University of Technology, Roosevelta Str. 40, 41-800 Zabrze, Poland; marta.kiel-jamrozik@polsl.pl; 4 Department of Mechatronics and Mechanical Systems Engineering, Polytechnic School of Engineering, University of Sao Paulo (USP), São Paulo 05508-900, SP, Brazil; gfbatalh@usp.br

**Keywords:** welding, neural network, virtual sensor, thermography, hardness, TIG

## Abstract

Maintaining high-quality welded connections is crucial in many industries. One of the challenges is assessing the mechanical properties of a joint during its production phase. Currently, in industrial practice, this occurs through NDT (non-destructive testing) conducted after the production process. This article proposes the use of a virtual sensor, which, based on temperature distributions observed on the joint surface during the welding process, allows for the determination of hardness distribution across the cross-section of a joint. Welding trials were conducted with temperature recording, hardness measurements were taken, and then, neural networks with different hyperparameters were tested and evaluated. As a basis for developing a virtual sensor, LSTM networks were utilized, which can be applied to time series prediction, as in the analyzed case of hardness value sequences across the cross-section of a welded joint. Through the analysis of the obtained results, it was determined that the developed virtual sensor can be applied to predict global temperature changes in the weld area, in terms of both its value and geometry changes, with the mean average error being less than 20 HV (mean for model ~35 HV). However, in its current form, predicting local hardness disturbances resulting from process instabilities and defects is not feasible.

## 1. Introduction

Welding plays a pivotal role in the industrial sector, serving as the backbone of manufacturing, construction, and engineering projects around the globe. It provides a robust method for joining metals and thermoplastics, enabling the creation of complex structures and machinery essential for infrastructure development, transportation networks [1], and energy production [2]. The versatility and strength of welding techniques facilitate innovations in design and materials science, driving advancements in aerospace, automotive, and renewable energy industries. Furthermore, the continual evolution of welding technologies, including automation and robotic welding, underscores its critical importance in enhancing efficiency, safety, and quality in industrial operations. As such, welding remains an indispensable skill and process, underpinning the growth and sustainability of modern industrial economies. Inconel is a trademark for a group of metal alloys known for their high nickel–chromium content and exceptional heat resistance, making them ideal for extreme environments such as jet engines and gas turbines [3]. Additionally, Inconel superalloys exhibit excellent resistance to corrosion and creep, ensuring long-term durability and reliability in harsh operating conditions. Maintaining those properties during welding as well as repairing using welding techniques are vital tasks, and they have been widely investigated [4,5,6,7].

The fundamental principle behind using artificial neural networks (ANNs) in welding quality monitoring is their ability to learn from data. By analyzing numerous parameters associated with the welding process, such as temperature, voltage, current, and feed rate, ANNs can identify complex relationships between these variables and the quality of the weld. This capability allows for the prediction of weld quality outcomes with high accuracy, even in the presence of variability in input parameters or environmental conditions. In the realm of research incorporating ANNs into welding practices, the advent of the error back-propagation algorithm in the mid-1980s marked the beginning of the ANN’s extensive application across various welding methodologies. This includes utilizing ANNs for predicting the quality of welds based on control parameters, recommending optimal welding control parameters to achieve specific weld characteristics, deriving profiles from laser vision sensing images, and employing vision sensors for quality and seam tracking in welding processes [8,9,10,11,12,13,14,15,16,17,18]. Furthermore, machine learning techniques have been extensively adopted in diverse welding procedures. These applications encompass leveraging deep learning and reinforcement learning for controlling laser welding, utilizing deep learning for the prediction of weld quality in arc welding, and implementing Support Vector Machine (SVM), a specific machine learning algorithm, for the classification of weld quality [11,12,13,14].

The prediction of weld mechanical properties based on temperature measurements using machine learning techniques represents a groundbreaking approach in the field of welding research and quality control. By leveraging the capabilities of machine learning algorithms, such as regression models, neural networks, or decision trees, researchers and engineers can accurately forecast the mechanical properties of welds, such as tensile strength, hardness, and ductility, from temperature data collected during the welding process. This method hinges on the principle that the thermal history of a weld zone is intricately linked to its microstructural evolution, which in turn dictates the mechanical properties of the weld. Machine learning models are trained on datasets comprising temperature profiles and corresponding mechanical properties from numerous welding experiments. Once trained, these models can predict the mechanical outcomes of new welds based solely on their thermal signatures. This predictive modeling offers immense potential for optimizing welding parameters in real time to achieve desired mechanical properties, significantly enhancing the efficiency and reliability of welding operations. It also minimizes the need for costly and time-consuming post-weld mechanical testing, thereby streamlining the manufacturing process. The use of machine learning for predicting weld mechanical properties from temperature measurements is a vivid illustration of how digital technologies are transforming traditional manufacturing practices, paving the way for smarter, more adaptable, and efficient production systems.

The knowledge about the welding process state and, finally, about the quality and properties of joints is based on observations and measurements using different type of sensors. A physical sensor responds to physical inputs such as temperature, noise, vibrations, or light intensity, converting these into impulses, usually in the form of electrical signals that can be digitized and recorded. In contrast, a virtual sensor, which operates solely on software, autonomously generates outputs by synthesizing and integrating data received (either synchronously or asynchronously) from physical sensors or other virtual sensors [15]. Virtual sensors exclusively utilize data collected by physical sensors. The information they produce is commonly incorporated into more sophisticated functions or software applications. These applications amalgamate this input with data from additional sources and apply analytical algorithms to the aggregated dataset [16,17]. These sensors are favored because they are adaptable, cost-effective, and capable of measuring elements that are challenging to assess due to their complexity [16]. In welding practice, virtual sensors are applied to predict weld bead geometry based on process parameters [18], the detection of wear of the secondary cable of a welding gun [19], and the control of robotic welding [20].

In the case of the welding process and the prediction of mechanical properties like hardness or impact strength, the main drawback limiting the possibility of the elaboration and application of these methods is the necessity of gathering a relatively large amount of real-life data. This demands the performance of many welding trials with different process parameters to obtain samples with different qualities of joints [21,22]. Next, proper analysis must be made, which is preceded with the cutting of samples, etching, etc. [23,24,25]. This high workload cannot be omitted by sample generation using, e.g., finite-element-method simulation. This is the case because in simulations for fixed sets of parameters, the same results will always be obtained [26,27,28]. During welding for the same set of parameters, slightly different results are gathered due to differences in the material structure or temporary changes in process conditions [29,30,31].

In this paper, the design and application of a novel virtual sensor that can be used to predict changes in hardness in the cross-section of a welded joint is described. The key idea was to apply temperature profiles gathered on the surface of a welded workpiece in three different distances of the prediction area (under the welding torch). The main innovation is the transformation from a two-dimensional temperature matrix into a hardness vector, which can be used directly during manufacturing to evaluate local faults of welding that lead to disturbance or the lowering of joint quality and mechanical properties.

## 2. Materials and Methods

**Figure 1:**
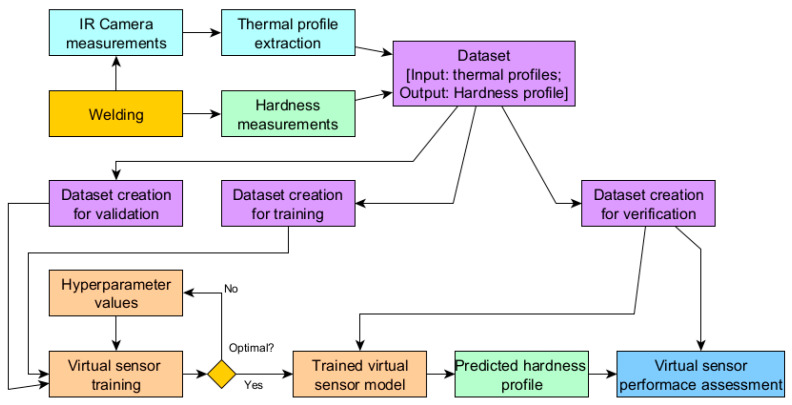
Diagram of performed research stages.

To develop a virtual sensor of acceptable quality, several steps must be taken (Figure 1). Initially, an active experiment should be conducted where joints are produced under various process settings. Throughout this process, the temperature distribution on the surface of the workpiece samples is recorded. In the subsequent step, the samples are sectioned, grouped into sets for each, and secured through hot mounting. Following this, the specimens are subjected to grinding and polishing to achieve a high-quality surface, making them suitable for hardness testing. The hardness of each sample is determined using the Vickers hardness test. The collected hardness data are then linked with the corresponding thermal profiles. Ultimately, this dataset is utilized to develop an artificial neural network designed to forecast the hardness distribution across the cross-section of welded joints.

### 2.1. Welding of Test Joints

The test samples, crafted from the nickel superalloy Inconel 600, were subjected to TIG welding and were all fabricated from 1.2 mm thick sheets. These materials were procured from Huntington Alloys Corporation located in the Huntington, WV, USA. The creation of this material entailed an industrial procedure, which included melting the Inconel 600 inside an electric furnace. Following the melting process, the material underwent plastic deformation through cold rolling, which was intermittently coupled with a heat treatment process known as recrystallization annealing. The chemical composition of the used material is shown in Table 1.

**Figure 2:**
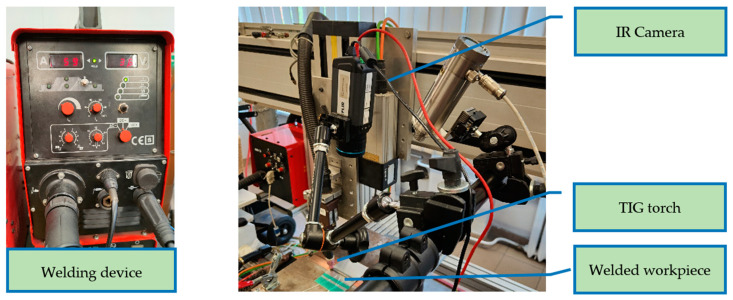
Welding device, TIG torch, and IR camera used during studies.

All workpieces were joined using the Casto TIG 2002 device (Castolin Eutectic Gmbh, Kriftel, Germany, Figure 2). The TIG welding of thin sheets was performed under laboratory conditions with the following constant parameters: shield gas flow Ar 12 L/min, ridge shield gas flow Ar 3 L/min. For welding a tungsten electrode (thoriated), type WT20 (diameter 2.4 mm) was used.

The temperature distribution across the welded joints was captured using an FILR A655sc infrared camera, as shown in Figure 1. This camera boasted a spatial resolution of 640 × 480 pixels and a 16 bit dynamic range and was calibrated with an emissivity setting of ε = 0.13. The change in emissivity related to temperature variation was disregarded for the methodologies applied, as there was no requirement to differentiate temperatures between the molten and solidified areas of the joint. Additionally, any reflections emanating from the intense heat of the welding torch and the welding arc itself were adjusted for using a fixed reflected temperature value. Infrared images were recorded at a frame rate of 60 fps. The optical axis of the camera was positioned at an 87-degree angle to the sample’s plane, maintaining a 600 mm gap from the welded object. Data post-processing and analysis was performed in the MATLAB R2023b environment with the use of an additional Teledyne FLIR Science File SDK (to read and process IR sequences).

**Figure 3:**
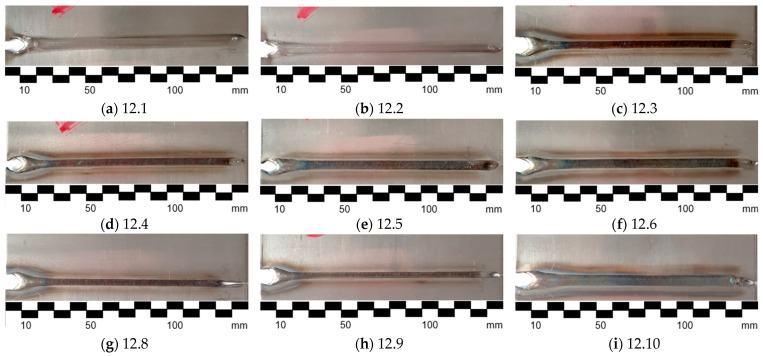
Welded samples made during the experiment. The side of the weld face is shown.

Joints were made for various sets of process parameters, which are shown in Table 2. The selection of parameters was conducted in order to achieve joints differing in mechanical properties. The resulting joint face is presented in Figure 3. It can be seen that the seam width as well as the discoloration of welds is different for various sets of parameters that are causing different amounts of heat input.

### 2.2. Hardness Examination

**Figure 4:**
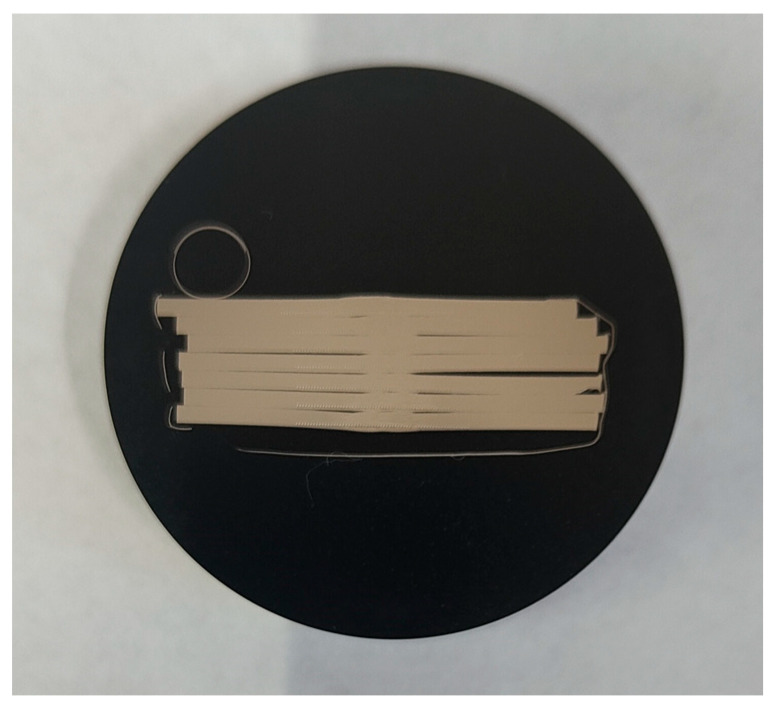
Package of samples after inclusion in self-hardener resin. Samples extracted from workpiece no. 12.2.

To examine the joint zone (JT), the heat-affected zone (HAZ), and the base material area (BM), Adler’s reagent was applied to the joint surfaces. This etchant is composed of 3 g of ammonium chlorocuprate ((NH_4_)_3_(CuCl_4_)), 20 mL of distilled water, 50 mL of hydrochloric acid (HCl), and 15 g of iron chloride (FeCl_3_). The etching process was performed incrementally, lasting between 10 and 15 s at ambient temperature. Macro-level examination of the etched joints was conducted with an OLYMPUS GX71 light microscope by Olympus Corporation (Tokyo, Japan), featuring up to 50× magnification. The breadth of the HAZ and the dimensions of the welded joint’s face and ridge were accurately measured to within ±0.1 mm using metallographic analysis under a microscope. Metallographic examinations at the microscopic level involved analyzing welded joint samples cut across the weld’s longitudinal axis. These samples were set in Duracryl Plus self-hardening resin (Figure 4), then sequentially sanded on water-based abrasive sheets ranging from 320 to 1000 grit. With each switch in abrasive paper, the sanding direction was altered by 90°. The samples were then polished on a Struers Planopol-3 machine (Struers A/S, Ballerup, Denmark) using a felt disk and an Al_2_O_3_ water suspension. The hardness of the cross-sectional areas was measured employing the Struers DuraScan 50 hardness tester (Henri Hauser AG, Biel, Switzerland) with a force of 9.807 N; thus, the hardness of 1 HV (or HV) was measured.

### 2.3. Data Processing and Neural Model Elaboration

**Figure 5:**
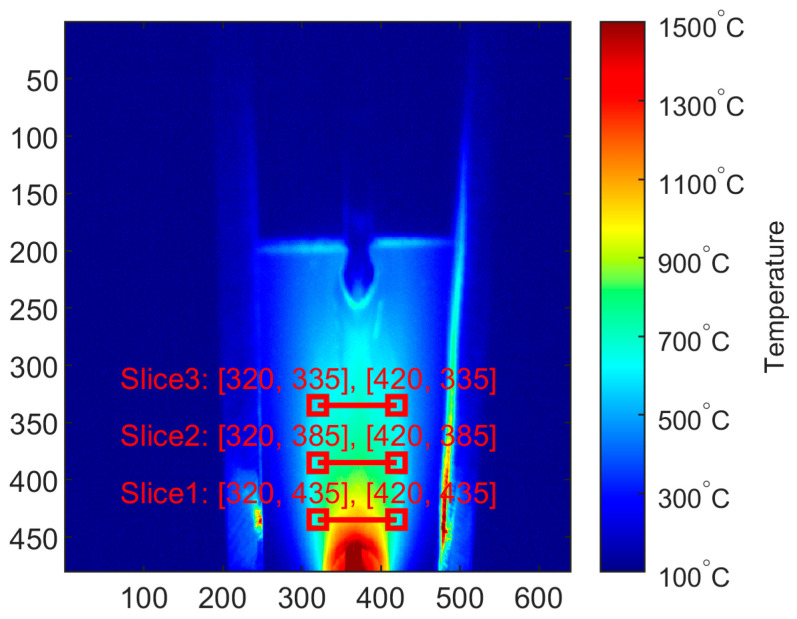
Thermogram taken during welding process of sample 12.1. With red lines, horizontal profiles are marked. From this IR image and corresponding hardness profile, training pair no. 12.1.2 was crated.

The first stage that influences results is the extraction of temperature profiles. For test purposes, three profiles were extracted, and the offset between them was 50 px. Due to perspective distortion, profiles were cut out in the bottom area of the thermogram, which represented the region closer to the welding torch. In this region, geometric distortion was low, and the image was in focus. As there was a constant welding speed set on the automated weld stand (Figure 2), there was a linear relationship between welding speed and the position of the welded workpiece. Additionally, thermograms were recorded with a constant framerate; thus, an IR image where the workpiece was in an assumed position in relation to the reference point was developed. Thanks to these dependencies, thermograms were found, in which the lowest profile (Slice 1, Figure 5) was in the approximate location at which the workpiece was cut apart to create samples for hardness measurements.

Extracted profiles, three for each image, were linked with corresponding hardness profiles. Thus, a pair of input data to virtual sensor and expected sensor output was created.

**Figure 6:**
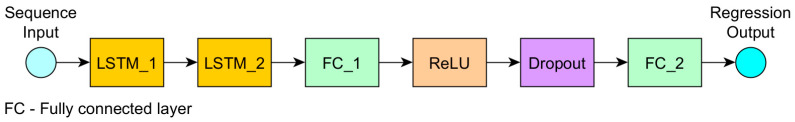
Schematic diagram of neural network used as a virtual sensor for hardness prediction.

To find the best combination of networks’ topologies and hyperparameters, the MATLAB Experiment Manager was used. The general structure of the network is presented in Figure 6. The network consists of LSTM Layers (one or two), a fully connected layer (FC), a rectified linear unit layer (ReLU), and a dropout layer. The LSTM architecture includes cell states and gates (input, forget, and output gates) that control the flow of information, allowing the network to retain or discard information over long sequences. The network’s ability to selectively remember and forget information allows it to capture intricate patterns and relationships in the sequence data. The ReLU layer is used to introduce non-linearity into the model, while the dropout layer realizes a regularization task that is used to prevent overfitting.

Three parameters were optimized during one experiment session: LSTM network depth, number of hidden units, and initial learning rate. LSTM network depth is a parameter that directly influences the topology of the network. After the input layer, a number of LSTM layers is present. The first experiment was a preliminary one. In this one, only the suitability of the general network topology was checked, without judging the values of creatin parameters. The number of layers was optimized and the best value was searched for in a range from one layer to seven layers in experiment 2 and [1; 2] in experiment 3, respectively. The number of LSTM layers affects a model’s capacity to learn hierarchical patterns and long-term dependencies, with more layers enabling deeper feature extraction but increasing the risk of overfitting and computational complexity. Balancing the number of layers is crucial for achieving optimal performance without excessive overfitting or excessive training time. The next hyperparameter that was optimized was the number of hidden units in each LSTM layer. In this case, the search range was [10; 600] in experiment 2 and [1; 900] in experiment 3. The number of hidden units determines the capacity of a model to learn and represent complex patterns in the data. It also affects the model’s ability to generalize from training data to unseen data and influences the ability of the LSTM to retain and utilize information over long sequences. The initial learning rate range was [0.001; 0.3] in both experiments. The initial learning rate is crucial for determining the convergence speed, stability, and final performance of an LSTM network; a high learning rate can lead to fast but potentially unstable convergence, while a low learning rate results in slower, more precise training. A wider range of parameters in the case of the second experiment was chosen to check a field of possible solutions. Then, the final, optimal network was selected using only a limited range of parameters in which the previous best ones were placed.

## 3. Results and Discussion

**Figure 7:**
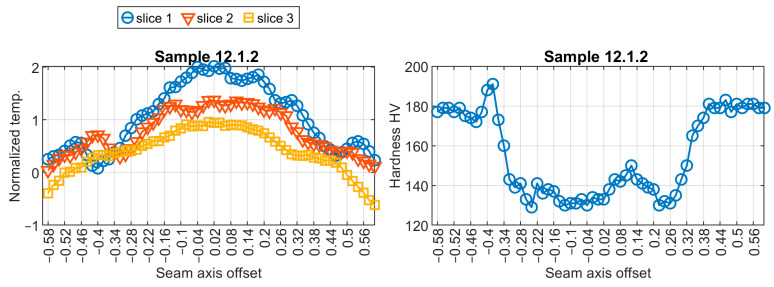
Visualization of training data pair no. 12.1.2.

**Figure 8:**
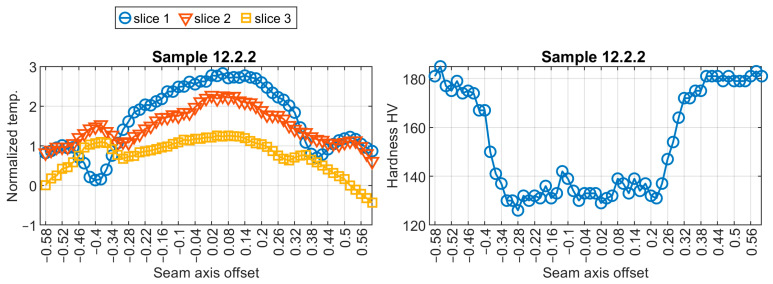
Visualization of training data pair no. 12.2.2.

The elaboration of the neural model for a virtual sensor started with training pairs’ extraction. There were 93 hardness profiles measured for nine welded test pieces. Corresponding thermal profiles were found in the sequences of thermograms taken during the welding. Exemplary temperature profiles and resulting hardness profiles are presented in Figure 7 and Figure 8. For further calculations, temperature was standardized using the z-score distance measure. It can be noticed that slice 1, representing temperature profile, which is located closest to the welding pool, has the shape that is most relevant in the context of temperature changes in the joint.

After analyzing the results from all performed experiments (see Appendix A), the two best models were selected according to MAE. The detailed results for the two best models, N30e3 and N04e3, and for the reference model that gave weak results (N33e2) are shown in Table 3. It can be noticed that there were quite large differences between hardness profile prediction quality within one model for various welded samples. For example, for N30e3, which was considered as the best model, the MAE varied from 24.72 to 47.54. Moreover, this model was not the best choice for all test samples, as for 12.2.5 and 12.5.2, N04e3 performed better according to MAE. Because of this, both models were further evaluated in detail.

The structure of the N30e3 network is presented in Table 4. The network had eight layers and 8.7 million learnable parameters in total. In contrast, the N04e3 network was smaller and had 7 million learnables. The training time of each individual network was similar for all models, and it was in the range of one to two minutes.

**Figure 9:**
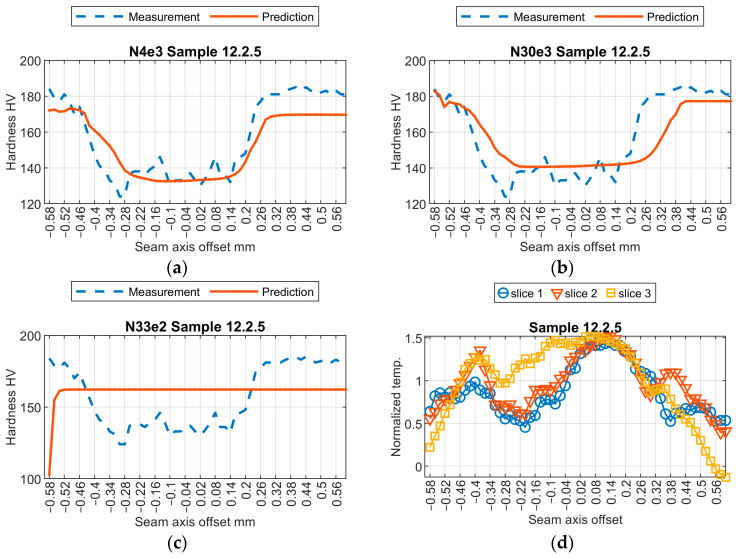
Results of virtual sensor testing for sample no. 12.2.5: (**a**) model N04e3; (**b**) model N30e3; (**c**) model N33e2; (**d**) input data.

**Figure 10:**
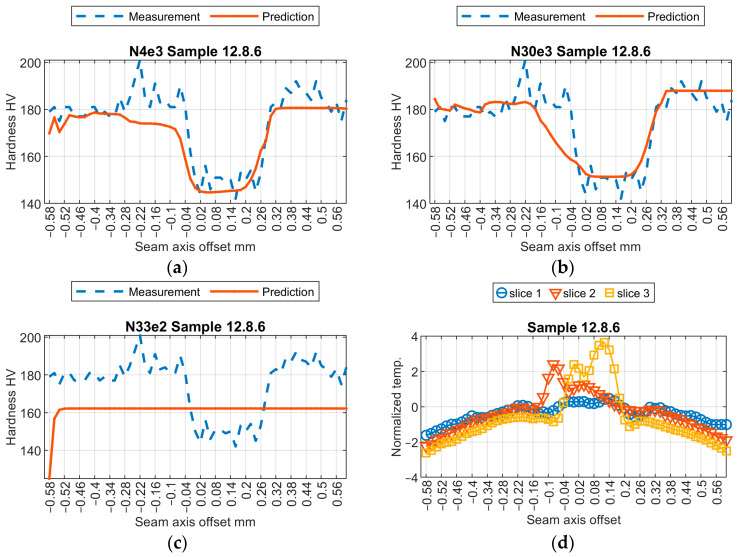
Results of virtual sensor testing for sample no. 12.8.6: (**a**) model N04e3; (**b**) model N30e3; (**c**) model N33e2; (**d**) input data.

**Figure 11:**
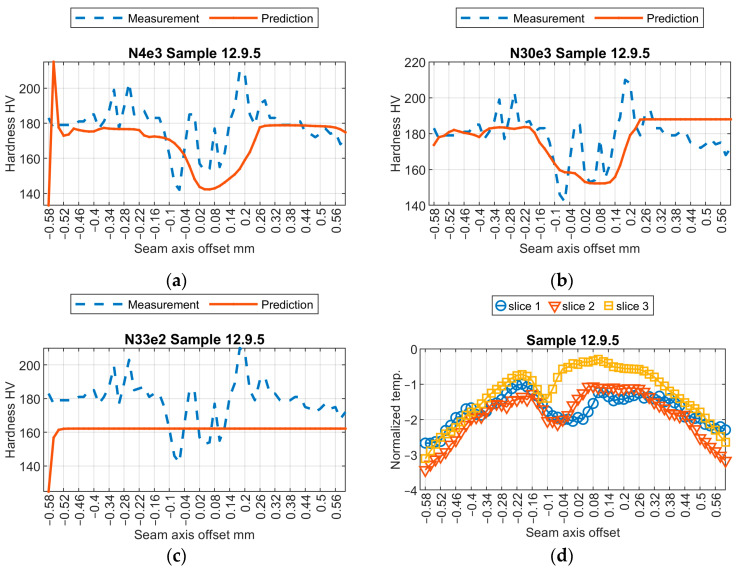
Results of virtual sensor testing for sample no. 12.9.5: (**a**) model N04e3; (**b**) model N30e3; (**c**) model N33e2; (**d**) input data.

**Figure 12:**
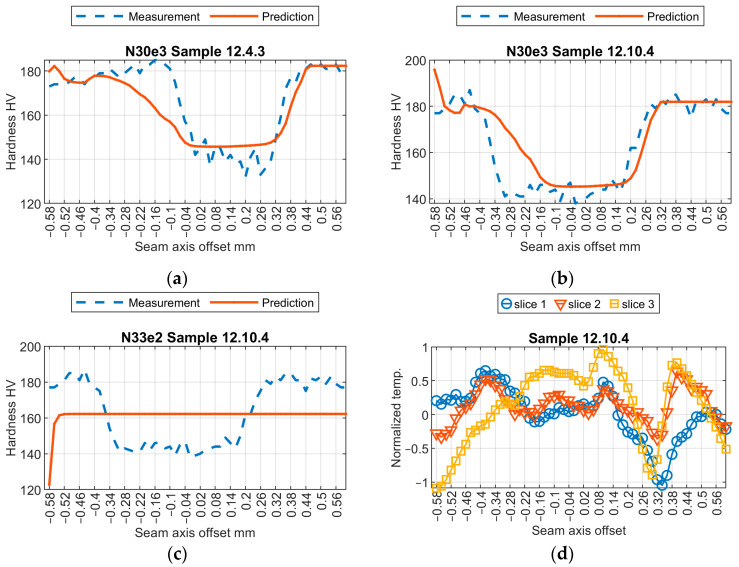
Results of virtual sensor testing for sample no. 12.10.4: (**a**) model N04e3; (**b**) model N30e3; (**c**) model N33e2; (**d**) input data.

Initially, a subjective assessment of the results obtained from three selected models was conducted. Relevant results from the samples are shown in Figure 9, Figure 10, Figure 11 and Figure 12. The first observed phenomenon concerns the shape of the obtained hardness profiles. In the case of the N04e3 models, the better representation of transition zones, where a decrease in hardness values occurs before the weld and then increases, was observed in most cases. On the other hand, regarding the representation of hardness values in the weld zone, the responses obtained from the N04e3 model are characterized by the better representation of minimum values. However, responses from the N30e3 model tend to indicate maximum values obtained in the weld area.

To quantify the similarity between models, additional metrics were used, as the subjective assessment of predicted shapes of hardness profiles differs from the objective one. This is especially the case when results seem to differ significantly after visual investigation, but the difference in the value of the mean maximal absolute error is low. In order to investigate the performance of the selected models, which are the core of virtual sensors, in more detail, several additional similarity measures were applied. The first are common distance measures: Euclidean distance and Manhattan distance [32]. Those measures allow one to measure the straight-line distance between two points in Euclidean space. They are simple approaches but might not be effective for time series that are shifted in time or amplitude. The next applied measure is the cosine similarity [33]. It measures the cosine of the angle between two vectors, which can be useful for understanding the similarity between two series regardless of their magnitude. Another simple and commonly used metric is the Pearson correlation [28]. It can be used to assess the similarity of vectors regardless of their average values and variability, but at the same time, this type of correlation assumes that the relationship is linear and that the data are normally distributed, which might not always be the case in real-world data. The longest common subsequence (LCSS) is a method used to measure similarity between two sequences by identifying the longest subsequence present in both sequences without changing the order of items [34]. This method is beneficial in problems where patterns over time/location are more important than the actual values of data points. The final approaches used are dynamic time warping (DTW) and restricted time warping [35]. DTW allows the stretching and compressing of data series to find an optimal match, making it more suitable for time series that may vary in speed. In the restricted version of DTW, additional constraints are applied on the warping window, limiting how far the alignment can stray locally. The purpose of this is to prevent extreme distortions in the time alignment, which might be irrelevant.

**Figure 13:**
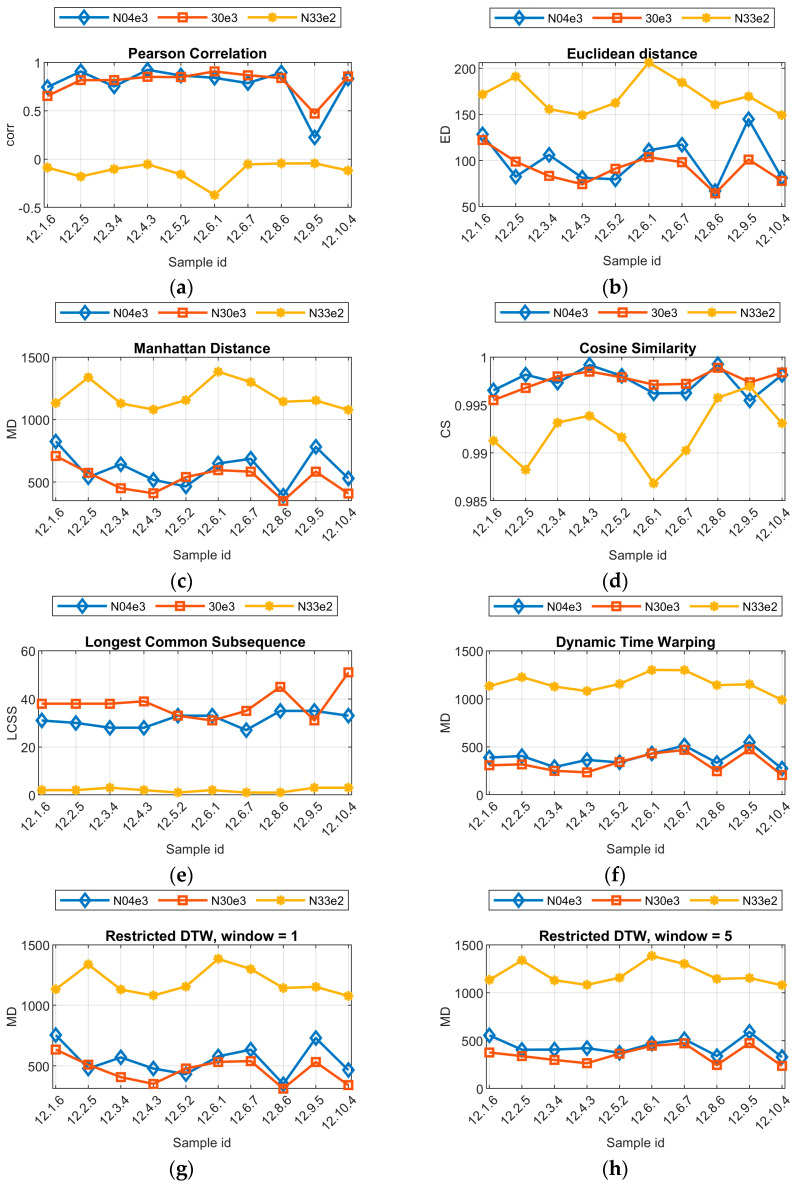
Similarity of real and predicted hardness profile measured with different metrices: (**a**) Pearson correlation; (**b**) Euclidean distance; (**c**) Manhattan distance; (**d**) cosine similarity; (**e**) longest common subsequence; (**f**) dynamic time warping (DTW); (**g**) restricted dynamic time warping, window length 1; (**h**) restricted dynamic time warping, window length 5.

Applying eight described measures led to a set of results (Table 5, Figure 13). First, it can be noticed that in most cases, the general order of model performances stands. Nevertheless, for the cosine similarity, the results for sample 12.9.5 (Figure 11) are opposite to those of the visual assessment of the hardness profile outputted by models. In this case, CS suggests that the results given by model N33e2 are better than the output from the N04e3 model. This is incorrect, because N04e3 results in a general shape and approximate value of hardness change in the joint area that is well reproduced, while the N33e2 model only produces a straight line. For other metrices, there is common disagreement when evaluating the quality of the N04e3 and N33e3 models. The comparison of LCSS and RDTW is especially interesting and gives insights into how models behave for certain data inputs. The LCSS measures more relevant positions, similar to DTW and RDTW. E.g., in the case of sample 12.10.4 (Figure 12), the large advantage of the N30e3 model is visible. It can be seen, when analyzing the plots of ground truth and predicted profiles, that the N30e3 model converges better with real data, as the left slope of the hardness decrease in the joint is closer to the real one, and for the right slope, where hardness increases, the offset between the real data and predicted data is smaller than that in the case of the N04e3 model. In DTW, where the translation of the hardness profile is less important than its shape, the N04e3 model is favorable, because the predicted hardness profile (and also the exact hardness values) is closer to the real data, mainly in the joint part, but it is translated. The property of LCSS, in which some points of profiles can remain unmatched, leads to intuitively better results in this case, because no correspondence has to be found for noisy or degraded regions.

## 4. Conclusions and Future Developments

This contribution aims to introduce the current state-of-the-art deep learning techniques to the prediction of hardness distribution on the cross-section of a welded joint. The prediction is based on a virtual sensor that utilizes information about the process temperature recorded on the workpiece surface. The proposed method was tested on real-life data obtained during an active welding experiment, where welded joints were produced for the same type of welded material using various process parameters. The conclusions obtained in this paper can be summarized in the following points:

- A method was developed to be applied to predict the hardness of joints made using TIG (GTAW—gas tungsten arc welding).
- The selected neural networks, being the bases of the virtual sensor, have two LSTM layers and 746 and 851 neurons in each layer, respectively.
- The best hardness profile prediction was made with N04e3. In this case, the MAE was 19.62 HV. The best overall performance was obtained for the N30e3 model, for which the average MAE for all verification samples was 34.12 HV.
- The application of profile similarity measurements allowed the in-depth investigation of virtual sensor performance. It was revealed that MAE global error is good for quantifying the overall quality of model output, but similarity measures like longest common subsequence (LCSS) and restricted dynamic time warping (RDTW) can also be successfully applied to quantify local quality, especially in the case of transient profile regions between joints and base materials.

The main question that arose at the stage of result analysis was whether the obtained results were suitable enough to indisputably state that joints can be accepted as meeting quality requirements in terms of mechanical properties. As the dataset used to train neural models was limited, the identification of hardness deviations, especially in the heat-affected zone, was insufficient. Local changes, especially increases in hardness within the heat-affected zone, remained unrepresented. Additionally, there were no local hardness changes in the fusion zone, which would manifest as temperature variations in the input profiles. On the other hand, the decrease in hardness in the fusion zone was accurately replicated (within the range of mean values). The width of the zone with reduced hardness was also effectively predicted.

Possible directions for further development are grouped into three main areas. First of all, additional samples have to be welded and new data acquired for different material geometries (e.g., the thickness of welded sheets). The authors assume that there can be a neural model that is specialized in assessing certain types of joints made from a single material or for a small group of related materials. The second direction is connected to the enhancement of model input space to allow signals from other sensors and process parameters to be used. The last considered development path is devoted to the further optimization of the neural model in terms of structure and other error or similarity measures that would be used to steer network training.

## Figures and Tables

**Table 1:** Chemical composition of the investigated Inconel superalloys.

Super- Alloy	Element Concentration, % wt													
	Ni	Cr	Fe	Mo	Nb	Co	Mn	Cu	Al	Ti	Si	C	S	P
Inconel 600	74.43	15.76	8.60	-	0.08	0.05	0.25	0.09	0.18	0.27	0.12	0.01	0.002	0.005
	Concentration proportion of elements							Nb + Ta—0.08%; Ni + Co—74.48%, Ta—0.0002%						

**Table 2:** TIG welding parameters used for the generation of samples.

ID	Plate Thickness mm	Current A	Welding Speed mm/s
12.1	1.2	60	3.0
12.2	1.2	60	3.0
12.3	1.2	60	5.0
12.4	1.2	60	5.0
12.5	1.2	60	4.0
12.6	1.2	60	4.0
12.8	1.2	60	7.0
12.9	1.2	60	7.0
12.10	1.2	70	4.0

**Table 3:** MAE results obtained for hardness prediction with use of two best models and reference model.

Model	Sample										Avg
	12.1.6	12.2.5	12.3.4	12.4.3	12.5.2	12.6.1	12.6.7	12.8.6	12.9.5	12.10.4	
N30e3	47.54	33.54	30.88	24.72	32.66	34.33	39.57	29.35	38.87	29.74	34.12
N04e3	52.03	19.62	31.62	24.16	29.62	41.53	49.15	26.92	55.99	22.60	35.32
N33e2	64.82	81.20	55.34	50.60	62.27	101.16	59.82	54.09	58.17	54.25	64.17

**Table 4:** Detailed structure of N30e3 network.

Name	Type	Learnable Sizes
SI	Sequential input, 3 dimensions	-
LSTM_1	LSTM with 851 units	Input weights 3404 × 3 Recurrent weights 3404 × 851 Bias 344 × 1
LSTM_2	LSTM with 851 units	Input weights 3404 × 851 Recurrent weights 3404 × 851 Bias 344 × 1
FC_1	100 fully connected layers	Weights 100 × 851 Bias 100 × 1
ReLU	ReLU	-
Dropout	50% dropout	-
FC_2	1 fully connected layer	Weights 1 × 100 Bias 1 × 1
RO	Absolute mean squared error	-

**Table 5:** Results of model assessment with different series similarity measures.

Model	Sample									
	12.1.6	12.2.5	12.3.4	12.4.3	12.5.2	12.6.1	12.6.7	12.8.6	12.9.5	12.10.4
Manhattan Distance										
N04e3	826	539	642	518	466	649	686	391	781	529
N30e3	709	574	451	410	540	595	583	350	584	408
N33e2	1133	1337	1129	1081	1155	1383	1299	1143	1152	1077
Cosine Similarity										
N04e3	0.9965	0.9982	0.9973	0.9992	0.9980	0.9962	0.9963	0.9993	0.9955	0.9981
N30e3	0.9955	0.9968	0.9980	0.9985	0.9979	0.9971	0.9972	0.9989	0.9974	0.9984
N33e2	0.9913	0.9883	0.9932	0.9939	0.9917	0.9868	0.9903	0.9958	0.9969	0.9931
Euclidean Distance										
N04e3	128	82	106	81	80	111	117	67	145	81
N30e3	122	99	83	74	91	104	98	64	101	78
N33e2	172	191	156	149	162	206	185	160	170	149
Longest Common Subsequence, Δ = 5										
N04e3	31	30	28	28	33	33	27	35	35	33
N30e3	38	38	38	39	33	31	35	45	31	51
N33e2	2	2	3	2	1	2	1	1	3	3
Pearson Correlation										
N04e3	0.7430	0.9050	0.7503	0.9220	0.8634	0.8396	0.7847	0.8932	0.2234	0.8320
N30e3	0.6518	0.8159	0.8154	0.8489	0.8463	0.9056	0.8663	0.8372	0.4696	0.8537
N33e2	−0.0883	−0.1799	−0.1032	−0.0539	−0.1588	−0.3719	−0.0551	−0.0458	−0.0442	−0.1186
Dynamic Time Warping										
N04e3	388	404	288	363	337	429	513	333	548	272
N30e3	307	317	249	234	342	430	467	245	473	205
N33e2	1133	1227	1129	1081	1155	1302	1299	1143	1152	989
Restricted Dynamic Time Warping, W = 1										
N04e3	754	479	570	479	433	579	634	350	730	466
N30e3	635	510	408	352	478	534	540	314	532	342
N33e2	1133	1337	1129	1081	1155	1383	1299	1143	1152	1077
Restricted Dynamic Time Warping, W = 5										
N04e3	554	404	406	420	373	469	514	339	590	327
N30e3	376	338	298	263	363	446	470	245	473	235
N33e2	1133	1337	1129	1081	1155	1383	1299	1143	1152	1077

## Data Availability

The data presented in this study are available on request from the corresponding author. The data are not publicly available because the authors do not wish to publish additional material.

## Appendix A

The detailed results of the training and validation of models with various hyperparameter values have been compiled in the appendix.

**Table A1:** Neural model training results achieved in experiment no. 2.

Trial	LSTM Depth	Number of Hidden Units	Initial Learning Rate	Training RMSE	Training Loss	Validation RMSE	Validation Loss	Mean Max Absolute Error
1	6	536	0.0019	31.43	493.86	20.97	219.83	43.28
2	7	135	0.0688	29.79	443.81	22.69	257.39	43.82
3	4	499	0.0786	33.82	572.04	28.47	405.29	54.32
4	3	225	0.0017	31.79	505.45	15.43	119.05	40.86
5	3	270	0.0018	31.51	496.51	16.52	136.41	51.16
6	7	506	0.0010	31.58	498.73	20.68	213.80	40.92
7	7	435	0.0010	31.93	509.74	21.60	233.22	54.33
8	3	203	0.0020	34.27	587.25	21.80	237.66	52.01
9	7	599	0.0010	31.59	498.87	22.89	262.08	52.86
10	7	13	0.1082	27.13	368.01	21.69	235.30	44.20
11	7	10	0.0214	30.80	474.39	23.21	269.26	53.60
12	2	223	0.2638	50.55	1277.78	22.25	247.45	42.28
13	4	59	0.2998	35.60	633.66	23.00	264.48	42.12
14	7	178	0.2985	38.37	736.17	23.57	277.89	45.24
15	2	14	0.1162	27.51	378.39	15.78	124.45	40.77
16	2	12	0.2993	30.51	465.48	23.54	277.08	48.56
17	3	138	0.0826	31.83	506.63	32.29	521.25	57.14
18	2	600	0.2958	41.85	875.88	22.57	254.76	43.01
19	7	600	0.1347	39.64	785.53	66.97	2242.28	97.55
20	6	21	0.2987	32.00	512.12	27.31	372.91	50.03
21	2	407	0.2995	39.56	782.46	25.38	322.14	50.04
22	4	535	0.0010	31.90	508.82	16.01	128.23	48.44
23	2	600	0.0284	35.99	647.59	22.32	249.19	44.59
24	2	521	0.0060	26.36	347.36	16.52	136.50	40.01
25	2	599	0.0032	28.18	397.18	14.89	110.90	36.87
26	2	600	0.0015	32.73	535.68	18.36	168.50	42.04
27	2	600	0.0045	30.58	467.58	18.70	174.93	38.34
28	2	600	0.0052	25.97	337.19	18.75	175.69	42.16
29	2	488	0.0024	31.13	484.40	15.98	127.73	40.96
30	5	10	0.0010	33.28	553.64	23.47	275.33	58.03
31	2	495	0.0060	26.31	346.21	16.62	138.07	36.60
32	2	467	0.0078	28.54	407.34	17.69	156.45	40.91
33	2	12	0.0010	30.45	463.63	23.82	283.76	63.44
34	7	106	0.0010	33.20	551.05	23.15	267.96	56.99
35	7	387	0.0209	32.36	523.60	24.07	289.71	43.84
36	2	558	0.0038	30.53	465.97	17.68	156.22	39.77
37	5	295	0.1451	39.06	763.01	48.28	1165.26	75.82
38	2	499	0.0199	31.38	492.43	22.90	262.25	43.97
39	3	35	0.0669	26.99	364.30	19.04	181.23	42.28
40	7	95	0.2963	55.29	1528.53	34.93	610.01	64.52
41	3	491	0.0050	29.40	432.07	16.13	130.09	40.13
42	7	171	0.0283	30.73	472.09	24.38	297.12	45.49
43	2	580	0.0033	31.44	494.24	17.65	155.71	42.65
44	2	58	0.1515	30.34	460.28	18.62	173.42	42.21
45	6	495	0.0035	29.47	434.32	19.47	189.46	41.13
46	5	426	0.0228	31.03	481.57	22.65	256.45	41.94
47	2	175	0.0059	28.98	419.82	14.48	104.86	37.16
48	7	44	0.0073	31.03	481.51	21.08	222.12	42.30
49	2	209	0.0048	30.37	461.29	16.30	132.86	38.01
50	7	304	0.0057	26.68	355.80	19.71	194.34	39.66

**Table A2:** Neural model training results achieved in experiment no. 3.

Trial	LSTM Depth	Number of Hidden Units	Initial Learning Rate	Training RMSE	Training Loss	Validation RMSE	Validation Loss	Mean Max Absolute Error
1	1	85	0.1787	28.53	407.03	17.98	161.64	43.80
2	2	67	0.0015	31.42	493.75	23.22	269.69	60.36
3	2	138	0.0830	34.28	587.60	25.88	334.98	53.39
4	2	764	0.0011	27.09	366.97	14.87	110.54	36.67
5	2	688	0.0193	35.12	616.67	20.79	216.17	45.90
6	2	807	0.2717	27.37	374.50	21.30	226.94	41.82
7	2	747	0.2323	40.38	815.43	20.13	202.70	42.25
8	2	899	0.0011	33.48	560.61	16.58	137.42	40.28
9	2	662	0.0010	31.17	485.89	14.24	101.42	37.75
10	1	59	0.0023	27.13	368.13	16.56	137.11	49.37
11	1	625	0.2939	55.65	1548.23	20.15	202.97	45.01
12	1	775	0.0010	26.76	357.95	16.64	138.48	55.67
13	2	742	0.0019	27.73	384.58	15.06	113.36	40.03
14	2	742	0.0010	30.31	459.27	15.89	126.30	39.82
15	2	737	0.0010	30.17	455.01	17.14	146.90	38.91
16	1	900	0.0482	32.65	532.89	23.76	282.36	48.17
17	1	424	0.0135	29.91	447.33	19.83	196.54	44.49
18	2	378	0.0010	32.64	532.66	16.77	140.67	42.87
19	2	602	0.0010	32.19	517.99	17.38	151.02	40.09
20	2	751	0.0010	34.19	584.34	15.79	124.72	39.47
21	1	382	0.0010	28.21	397.80	17.95	161.04	68.02
22	2	745	0.0014	31.98	511.46	15.23	115.98	36.84
23	2	900	0.0015	27.94	390.25	16.11	129.75	38.02
24	2	750	0.0013	30.22	456.71	15.15	114.78	38.39
25	1	15	0.0517	22.36	250.07	18.37	168.78	40.69
26	1	4	0.0234	23.91	285.87	17.81	158.67	40.14
27	1	272	0.0476	39.05	762.63	18.97	179.86	42.77
28	2	900	0.0034	25.69	329.96	15.47	119.59	37.06
29	2	900	0.0041	25.08	314.53	18.05	162.88	40.16
30	2	851	0.0020	27.55	379.50	13.74	94.46	35.79
31	2	853	0.0021	29.30	429.23	14.93	111.42	36.76
32	2	865	0.0021	25.62	328.20	14.23	101.25	37.88
33	2	15	0.2999	29.77	443.06	20.93	219.03	44.63
34	1	900	0.0062	29.39	431.88	15.78	124.45	38.63
35	2	899	0.0022	26.69	356.07	15.65	122.54	37.30
36	2	900	0.0777	37.14	689.83	23.05	265.70	49.53
37	1	898	0.0109	32.31	521.91	18.47	170.50	39.36
38	2	465	0.2998	31.46	494.72	20.26	205.18	42.06
39	2	899	0.0022	28.72	412.49	14.04	98.63	37.18
40	2	1	0.0120	27.91	389.58	16.80	141.15	39.76
41	2	898	0.0026	28.44	404.31	20.97	219.93	41.35
42	2	386	0.0083	29.69	440.84	18.48	170.74	42.51
43	2	899	0.0017	30.36	460.81	16.51	136.37	37.91
44	1	3	0.0079	26.76	358.16	17.12	146.62	41.20
45	1	899	0.1454	46.55	1083.58	35.68	636.40	61.21
46	1	581	0.0058	25.51	325.26	15.92	126.66	37.83
47	1	873	0.0038	27.88	388.78	16.77	140.67	41.64
48	1	710	0.0073	29.28	428.69	18.17	165.13	40.70
49	1	324	0.0050	29.75	442.67	18.97	179.90	44.51
50	2	1	0.0203	27.58	380.41	17.63	155.49	41.35

## References

[1] Chen H., Pinkerton A.J., Li L. (2011). Fibre laser welding of dissimilar alloys of Ti-6Al-4V and Inconel 718 for aerospace applications. Int. J. Adv. Manufact. Technol..

[2] Holub L., Dunovský L., Kovanda K., Kolařík L. (2015). SAW—Narrow Gap Welding CrMoV Heat-resistant Steels Focusing to the Mechanical Properties Testing. Procedia Eng..

[3] Pedroso A.F.V., Sousa V.F.C., Sebbe N.P.V., Silva F.J.G., Campilho R.D.S.G., Sales-Contini R.C.M., Nogueira F.R., Silva F.J.G., Pereira A.B., Campilho R.D.S.G. (2024). A Review of INCONEL^®^ Alloy’s Non-conventional Machining Processes. Flexible Automation and Intelligent Manufacturing: Establishing Bridges for More Sustainable Manufacturing Systems.

[4] Dinda G.P., Dasgupta A.K., Mazumder J. (2009). Laser Aided Direct Metal Deposition of Inconel 625 Superalloy: Microstructural Evolution and Thermal Stability. Mater. Sci. Eng. A.

[5] Ma D., Stoica A.D., Wang Z., Beese A.M. (2017). Crystallographic Texture in an Additively Manufactured Nickel-Base Superalloy. Mater. Sci. Eng. A.

[6] Sonar T., Balasubramanian V., Malarvizhi S., Venkateswaran T., Sivakumar D. (2021). An overview on welding of Inconel 718 alloy—Effect of welding processes on microstructural evolution and mechanical properties of joints. Mater. Charact..

[7] German C., Lin X. (2021). GTAW Welded Inconel 625 Alloy Fuel Cladding for the Canadian SCWR: Microstructure and Mechanical Property Characterization. ASME J. Nucl. Rad. Sci..

[8] Andersen K., Cook G.E., Karsai G., Ramaswamy K. (1990). Artificial Neural Networks Applied to Arc Welding Process Modeling and Control. IEEE Trans. Ind. Appl..

[9] Cook G.E., Barnett R.J., Andersen K., Strauss A.M. (1995). Weld Modeling and Control Using Artificial Neural Networks. IEEE Trans. Ind. Appl..

[10] Moon H.S., Na S.J. (1996). A Neuro-Fuzzy Approach to Select Welding Conditions for Welding Quality Improvement in Horizontal Fillet Welding. J. Manuf. Syst..

[11] Guenther J., Pilarski P.M., Helfrich G., Shen H., Diepold K. (2016). Intelligent Laser Welding Through Representation, Prediction, and Control Learning: An Architecture with Deep Neural Networks and Reinforcement Learning. Mechatronics.

[12] Kim M.S., Shin S.M., Kim S., Rhee D.H. (2018). A Study on the Algorithm for Determining Back Bead Generation in GMA Welding Using Deep Learning. J. Weld. Join..

[13] Petkovic D. (2017). Prediction of Laser Welding Quality by Computational Intelligence Approaches. Optik.

[14] Chen J., Wang T., Gao X., Wei L. (2018). Real-Time Monitoring of High-Power Disk Laser Welding Based on Support Vector Machine. Comput. Ind..

[15] Kabadayi S., Pridgen A., Julien C. Virtual sensors: Abstracting data from physical sensors. Proceedings of the International Symposium on a World of Wireless, Mobile and Multimedia Networks.

[16] Liu L., Kuo S., Zhou M. Virtual sensing techniques and their applications. Proceedings of the 2009 International Conference on Networking, Sensing and Control.

[17] Ko J., Lee B.-B., Lee K., Hong S.G., Kim N., Paek J. (2015). Sensor virtualization module: Virtualizing IoT devices on mobile smartphones for effective sensor data management. Int. J. Distrib. Sens. Netw..

[18] Fernández-Zabalza A., Veiga F., Suárez A., López J.R.A. (2024). The Use of Virtual Sensors for Bead Size Measurements in Wire-Arc Directed Energy Deposition. Appl. Sci..

[19] Ibáñez D., Garcia E., Soret J., Martos J. (2023). Incipient Wear Detection of Welding Gun Secondary Circuit by Virtual Resistance Sensor Using Mahalanobis Distance. Sensors.

[20] Cederberg P., Olsson M., Bolmsjö G. (2002). Virtual triangulation sensor development, behavior simulation and CAR integration applied to robotic arc-welding. J. Intell. Robot. Syst..

[21] Górka J. (2014). Analysis of simulated welding thermal cycles S700MC using thermal imaging camera. Mod. Technol. Ind. Eng..

[22] Kik T., Górka J., Kotarska A., Poloczek T. (2020). Numerical Verification of Tests on the Influence of the Imposed Thermal Cycles on the Structure and Properties of the S700MC Heat-Affected Zone. Metals.

[23] Jamrozik W., Górka J., Kik T. (2021). Temperature-Based Prediction of Joint Hardness in TIG Welding of Inconel 600, 625 and 718 Nickel Superalloys. Materials.

[24] Jamrozik W., Gorka J. (2021). Assessing MMA Welding Process Stability Using Machine Vision-Based Arc Features Tracking System. Sensors.

[25] Zhou X., Chen M., Shang W., Shen H., Xu H. Real time Monitoring Method of Welding Defects Based on LSTM Sequence Model. Proceedings of the 2023 IEEE 3rd International Conference on Information Technology, Big Data and Artificial Intelligence (ICIBA).

[26] Shang L., Zhang Z., Tang F., Cao Q., Yodo N., Pan H., Lin Z. (2023). Deep Learning Enriched Automation in Damage Detection for Sustainable Operation in Pipelines with Welding Defects under Varying Embedment Conditions. Computation.

[27] Luo Z., Wu D., Zhang P., Ye X., Shi H., Cai X., Tian Y. (2023). Laser Welding Penetration Monitoring Based on Time-Frequency Characterization of Acoustic Emission and CNN-LSTM Hybrid Network. Materials.

[28] Shi Y.H., Wang Z.S., Chen X.Y., Cui Y.-X., Xu T., Wang J.-Y. (2023). Real-time K-TIG welding penetration prediction on embedded system using a segmentation-LSTM model. Adv. Manuf..

[29] Li L., Cheng F., Wu S. (2021). An LSTM-based measurement method of 3D weld pool surface in GTAW. Measurement.

[30] Li H., Ma Y., Duan M., Wang X., Che T. (2023). Defects detection of GMAW process based on convolutional neural network algorithm. Sci. Rep..

[31] Kumaresan S., Aultrin K.S.J., Kumar S.S., Anand M.D. (2021). Transfer Learning With CNN for Classification of Weld Defect. IEEE Access.

[32] Chang F., Zhou G., Ding K., Li J., Jing Y., Hui J., Zhang C. (2023). A CNN-LSTM and Attention-Mechanism-Based Resistance Spot Welding Quality Online Detection Method for Automotive Bodies. Mathematics.

[33] Cassisi C., Montalto P., Aliotta M., Cannata A., Pulvirenti A. (2012). Similarity Measures and Dimensionality Reduction Techniques for Time Series Data Mining. Advances in Data Mining Knowledge Discovery and Applications.

[34] Li B., Han L., Yin H., Tang K., Gao Y., Klawonn F., Lee M., Weise T., Li B., Yao X. (2013). Distance Weighted Cosine Similarity Measure for Text Classification. Intelligent Data Engineering and Automated Learning—IDEAL 2013. IDEAL 2013. Lecture Notes in Computer Science.

[35] Bergroth L., Hakonen H., Raita T. A survey of longest common subsequence algorithms. Proceedings of the Seventh International Symposium on String Processing and Information Retrieval (SPIRE).

